# Improvement of the Diagnosis of Isoattenuating Pancreatic Carcinomas by Defining Their Characteristics on Contrast Enhanced Computed Tomography and Endosonography with Fine-Needle Aspiration (EUS-FNA)

**DOI:** 10.3390/diagnostics11050776

**Published:** 2021-04-26

**Authors:** Robert Psar, Ondrej Urban, Marie Cerna, Tomas Rohan, Martin Hill

**Affiliations:** 1 Faculty of Medicine and Dentistry, Palacký University Olomouc, 775 15 Olomouc, Czech Republic; 2 Department of Radiology, Vitkovice Hospital, 703 00 Ostrava-Vitkovice, Czech Republic; 3 AGEL Research and Training Institute, 796 04 Prostejov, Czech Republic; 4 Department of Internal Medicine II—Gastroenterology and Geriatrics, Faculty of Medicine and Dentistry, Palacký University Olomouc and University Hospital Olomouc, 775 15 Olomouc, Czech Republic; 5 Department of Radiology, Faculty of Medicine, University Hospital Olomouc, Dentistry Palacký University Olomouc, 779 00 Olomouc, Czech Republic; marie.cerna@fnol.cz; 6 Department of Radiology and Nuclear Medicine, University Hospital Brno and Masaryk University Brno, 625 00 Brno, Czech Republic; rohan.tomas@fnbrno.cz; 7 Institute of Endocrinology, 116 94 Prague, Czech Republic; mhill@endo.cz

**Keywords:** computed tomography, early diagnosis, endoscopic ultrasound-guided fine needle aspiration, isoattenuation, pancreatic carcinoma

## Abstract

(1) Background. The aim was to define typical features of isoattenuating pancreatic carcinomas on computed tomography (CT) and endosonography and determine the yield of fine-needle aspiration endosonography (EUS-FNA) in their diagnosis. (2) Methods. One hundred and seventy-three patients with pancreatic carcinomas underwent multiphase contrast-enhanced CT followed by EUS-FNA at the time of diagnosis. Secondary signs on CT, size and location on EUS, and the yield of EUS-FNA in isoattenuating and hypoattenuating pancreatic cancer, were evaluated. (3) Results. Isoattenuating pancreatic carcinomas occurred in 12.1% of patients. Secondary signs of isoattenuating pancreatic carcinomas on CT were present in 95.2% cases and included dilatation of the pancreatic duct and/or the common bile duct (85.7%), interruption of the pancreatic duct (76.2%), abnormal pancreatic contour (33.3%), and atrophy of the distal parenchyma (9.5%) Compared to hypoattenuating pancreatic carcinomas, isoattenuating carcinomas were more often localized in the pancreatic head (100% vs. 59.2%; *p* < 0.001). In ROC (receiver operating characteristic) analysis, the optimal cut-off value for the size of isoattenuating carcinomas on EUS was ≤ 25 mm (AUC = 0.898). The sensitivity of EUS-FNA in confirmation of isoattenuating and hypoattenuating pancreatic cancer were 90.5% and 92.8% (*p* = 0.886). (4) Conclusions. Isoattenuating pancreatic head carcinoma can be revealed by indirect signs on CT and confirmed with high sensitivity by EUS-FNA.

## 1. Introduction

Pancreatic carcinoma is a cancer with an extremely poor prognosis. In 2018, 458,918 new cases of pancreatic cancer, and up to 432,242 new deaths, were reported worldwide [1]. Among radically operated patients with pancreatic carcinoma, the five-year survival rate is only 4–34%, with a median survival of 17–27 months [2].

Computed tomography (CT) has become the method of choice in diagnosis, staging, treatment planning and monitoring of patients with pancreatic carcinoma. According to an extensive meta-analysis, reported sensitivity and specificity of CT in the diagnosis of pancreatic cancer is 90% and 87% [3]. CT has less benefit in pancreatic tumors smaller than 2 cm [4,5]. It is also known that at a tumor size of 2 cm, over 50% of patients have micrometastases [6].

Using contrast-enhanced CT, pancreatic carcinoma usually becomes hypoattenuating in the pancreatic (arterial) and venous phase. In isoattenuating carcinoma, the decrease of the tumor density compared to the surrounding parenchyma is usually less than 15 HU, and, therefore, generally imperceptible to the naked human eye on CT [7,8]. The presence of isoattenuating pancreatic carcinoma can be diagnosed by CT in the presence of so-called secondary signs. These most often include dilatation or interruption of the pancreatic duct, dilatation of the common bile duct (CBD), atrophy of the distal parenchyma and abnormal contour of the pancreas [7,8,9,10].

Histologically, isoattenuating pancreatic carcinomas are characterized by lower tumor cellularity, more frequent association with intratumoral acinar tissues and islet cells, and less frequent tumor necrosis, compared to hypoattenuating pancreatic carcinomas [8]. It is uncertain whether isoattenuating pancreatic carcinoma is a separate subtype of pancreatic cancer or a temporary early form of usual hypoattenuating pancreatic carcinoma [7,8]. On the other hand, isoattenuating pancreatic carcinoma have been shown to have much higher postoperative survival than hypoattenuating pancreatic carcinoma [7,8,9,10].

It is, therefore, very important to diagnose these isoattenuating tumors correctly and early.

Endoscopic ultrasound with fine needle aspiration (EUS-FNA) is considered to be the most accurate method in the diagnosis of pancreatic tumors. It made a major contribution to the verification of pancreatic cancer before neoadjuvant and palliative oncological treatment [11,12]. The limitation of EUS-FNA is the risk of false negativity and thus low negative predictive value. Therefore, negative cytology does not reliably rule out pancreatic cancer [13,14].

There are few studies dealing with isoattenuating pancreatic carcinomas [8,9,10,15,16,17]. Of the other imaging methods, so far only the sensitivity of magnetic resonance (MR) and PET (positron emission tomography)/CT has been demonstrated in isoattenuating pancreatic carcinomas. MR showed isoattenuating pancreatic carcinoma in approximately 80% cases (19 of 24 patients) [8]. PET CT detected them with a sensitivity slightly lower than MRI, i.e., 73.7% [8]. To the best of our knowledge, the importance of EUS-FNA in the diagnosis of isoattenuating pancreatic carcinoma has not yet been evaluated.

The main goal of the study was to determine the possibility of diagnosing isoattenuating pancreatic cancer based on secondary signs on CT and to determine the sensitivity of EUS-FNA in the diagnosis of this tumor.

## 2. Materials and Methods

### 2.1. Study Design

**Figure 1:**
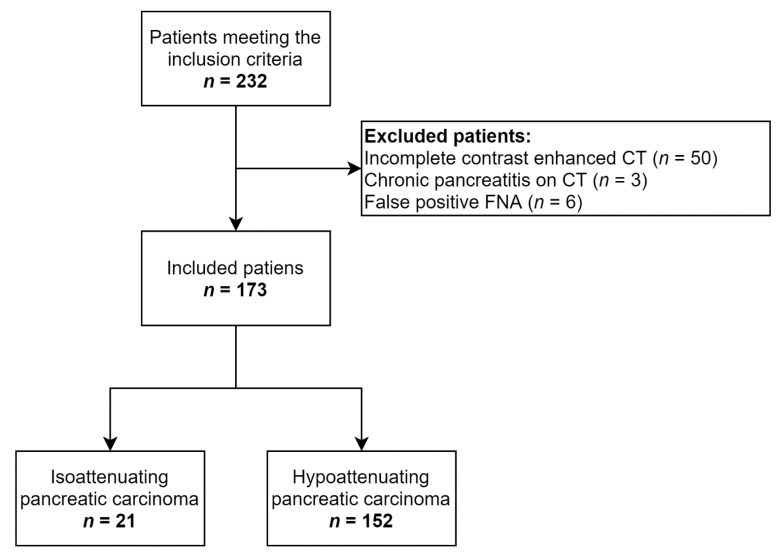
Flowchart demonstrating included patients.

The study was performed in a single tertiary referral center after approval by the institutional review board (number EK/15/2021). In this retrospective study were enrolled 232 consecutive patients with a definitive diagnosis of pancreatic carcinomas who underwent contrast-enhanced CT and consequent EUS-FNA at the time of diagnosis. In all patients, pancreatic carcinomas were visualized by EUS. Patients with evidence of severe advanced chronic pancreatitis (*n* = 3) [8,10], patients who underwent CT with no pancreatic or portal phase (*n* = 50), and patients with false positive EUS-FNA (*n* = 6), were excluded from the study. Finally, 173 patients were included in the study (Figure 1). No pancreatic calcifications or a pancreatic stent were present on diagnostic CT in any of the enrolled patients. Inclusion and exclusion criteria are listed in Table 1.

### 2.2. Gold Standard

Histology was the gold standard for the final diagnosis in operated patients. In patients without surgery, the diagnosis of pancreatic cancer was made on the basis of a typical radiographic and/or EUS-FNA findings and the concurrent clinical and radiographic progression of the disease during a one-year follow-up period.

### 2.3. EUS-FNA Examination Technique

EUS-FNA examination was performed by two expert endoscopists using a linear probe echoendoscope (Olympus GF UCT 140 AL, Olympus Europe, Hamburg, Germany) and a 22-Gauge FNA needle (EZShot NA-200H-8022, Olympus Europe, Hamburg, Germany). Evaluation of samples was performed with the participation of an experienced cytopathologist using the ROSE method (rapid on-site cytopathology evaluation). A detailed description is given in Appendix A.

### 2.4. CT Examination Technique

The pancreatic CT protocol included an initial unenhanced helical CT scan followed by a helical scan after administration of 80–100 mL of nonionic contrast material (Iopamirone 370 mg/mL, Bayer Schering Pharma, Osaka, Japan or Omnipaque 300 mg/mL, Daiichi Sankyo, Tokyo, Japan) at a rate of 3 mL/s by using an automatic injector. Postcontrast CT was performed in pancreatic parenchymal phase and portal venous phase (15–20 s after pancreatic phase). CT examination was performed on CTs with 16 and 128 rows of detectors (GE LightSpeed 16, GE Healthcare, Milwaukee, WI, USA; Ingenuity 128, Philips Healthcare, Amsterdam, The Netherlands). Detailed CT parameters are described in Appendix B.

### 2.5. Evaluation of CT Examination and Definitions of Secondary Signs of Pancreatic Carcinomas

Density and secondary signs of the tumor were retrospectively evaluated by CT independently by two radiologists (seven and 19 years of experience) in the DICOM (Digital Imaging and Communications in Medicine) viewer Intelispace Portal (Philips, Amsterdam, The Netherlands). In cases of disagreement between the radiologists, a consensus opinion was recorded. The pancreas was assessed separately in postcontrast pancreatic and portal venous phase images. The layer thickness was set to 3–5 mm, and the center and width of the CT window were 60 and 360 HU, respectively. The hypoattenuating and isoattenuating mass/suspicious areas were deemed as a tumor on contrast enhanced CT. Reported tumor size and location were not determined by CT, but by EUS, where the maximal diameter of the tumor was recorded.

If a lesion showed isoattenuation compared to surrounding pancreatic parenchyma during dynamic contrast-enhanced CT, it was considered isoattenuating pancreatic cancer [15]. The presumed localization of isoattenuating pancreatic carcinoma on CT was the area downstream from the interruption of the main pancreatic duct, dilated CBD or at the level of the abnormally contoured part of the pancreas [16]. In isoattenuating pancreatic carcinomas, the difference in the density of the suspected area and adjacent pancreatic parenchyma was less than 15 HU in both pancreatic and portal venous phases. The average value of density in the region of interest (ROI) of size 20–40 mm^2^ was recorded. In the area of the normal parenchyma, the ROI of the same size was placed as far as possible from the presumed lesion, omitting bile ducts, cysts and vessels to avoid incorrect densities [16].

CT scans were further retrospectively analyzed for the presence of secondary signs. Pancreatic duct interruption was defined as an abrupt luminal disruption of the main pancreatic duct with or without upstream duct dilatation. Pancreatic duct dilatation was determined as a maximum size of the main pancreatic duct > 3 mm [15]. Parenchymal atrophy was defined as atrophy distal to the tumor or as disproportionate atrophy distal to the presumed isoattenuating tumor. Bulging or loss of normal pancreatic lobulation was considered as an abnormal contour. Dilatation of the CBD was considered when the maximum diameter of the short axis of the CBD > 7 mm in patients younger than 60 years and ≥ 9 mm above 60 years [10].

### 2.6. Study Endpoints and Monitored Characteristics

The objective of the study was to determine the possibility of diagnosing isoattenuating pancreatic cancer based on secondary signs on CT and to determine the sensitivity of EUS-FNA in the diagnosis of this tumor. Followed characteristics on CT included pancreatic or CBD dilatation, interruption of pancreatic duct, pancreatic atrophy and abnormal contour of the pancreas. Furthermore, differences in age, gender, and frequency of surgical resection between iso- and hypoattenuating pancreatic cancer were analyzed.

### 2.7. Statistical Analysis

Differences in binary data were tested by Chi-squared test with Yates correction (EUS-FNA sensitivity, tumor location by EUS, sex, surgery and secondary signs seen on CT). Differences in size distribution on EUS were evaluated using a test of linear trend in proportions for ordinal data. The Mann Whitney robust test was used for evaluation of differences in metric data (tumor size on EUS and age). A *p*-value of < 0.05 was considered to be statistically significant. An ROC curve was performed to estimate CT isoattenuating pancreatic carcinomas based on their size measured by EUS.

## 3. Results

### 3.1. General Characteristics

Of the 173 patients (81 women) with a definitive diagnosis of pancreatic carcinomas, according to the EUS, a total of 111 (64.2%) were located in the head, 46 in the body (26.6%) and 16 in the pancreatic tail (9.2%). The diagnosis of pancreatic carcinoma was confirmed by surgical resection in 49.7% (86 of 173) of patients and by cytology during EUS-FNA in the remaining 50.3% (87 of 173) of patients. The median age of all patients was 68 years (range 40–88).

Isoattenuating pancreatic carcinomas occurred in 12.1% (*n* = 21) of patients, and hypoattenuating pancreatic carcinomas was observed in the remaining 87.9% (*n* = 152) of patients. No significant difference in age (median 70 vs 68 years, *p* = 0.993) or gender (61.9% vs 52.0% males, *p* = 0.534) between tumor groups was observed, while surgical resection with curative intent was significantly more frequent in isoattenuating carcinomas (85.7% vs 44.7%; *p* = 0.001). Basic characteristics are summarized in Table 2.

### 3.2. Secondary Signs of Isoattenuating Pancreatic Carcinomas on CT

**Figure 2:**
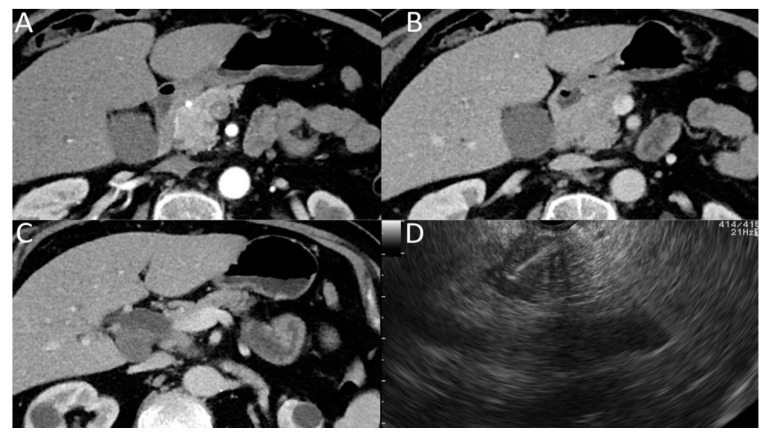
Isoattenuating pancreatic carcinoma on CT in pancreatic (**A**) and portal venous phase (**B**) with dilated common bile duct (**C**). EUS-FNA confirming the tumor of the head of the pancreas (**D**).

Secondary signs were present on CT in 95.2% (20 of 21) of patients with isoattenuating pancreatic carcinomas and included dilatation of the pancreatic duct or CBD (85.7%; Figure 2), interruption of the pancreatic duct (76.2%), abnormal contour of the pancreas (33.3%) and distal parenchymal atrophy (9.5%). The incidence of distal parenchymal atrophy (*p* ˂ 0.001) and abnormal pancreatic contour (*p* = 0.01) were significantly more common in hypoattenuating pancreatic carcinomas than in isoattenuating pancreatic carcinomas; differences in other described signs were insignificant. An overview of secondary features and statistical differences of isoattenuating and hypoattenuating pancreatic carcinomas is summarized in Table 3.

### 3.3. EUS Findings and the Role of EUS-FNA in the Confirmation of Pancreatic Cancer

On EUS, all isoattenuating pancreatic carcinomas were localized in the pancreatic head, which was significantly more often than in case of hypoattenuating pancreatic carcinoma (100% vs 59.2%; *p* < 0.001). All isoattenuating pancreatic carcinomas were significantly smaller in size compared to hypoattenuating pancreatic carcinomas (median 20 vs 31 mm; *p* < 0.001). No isoattenuating pancreatic carcinomas measured more than 30 mm on EUS. Table 4.

**Figure 3:**
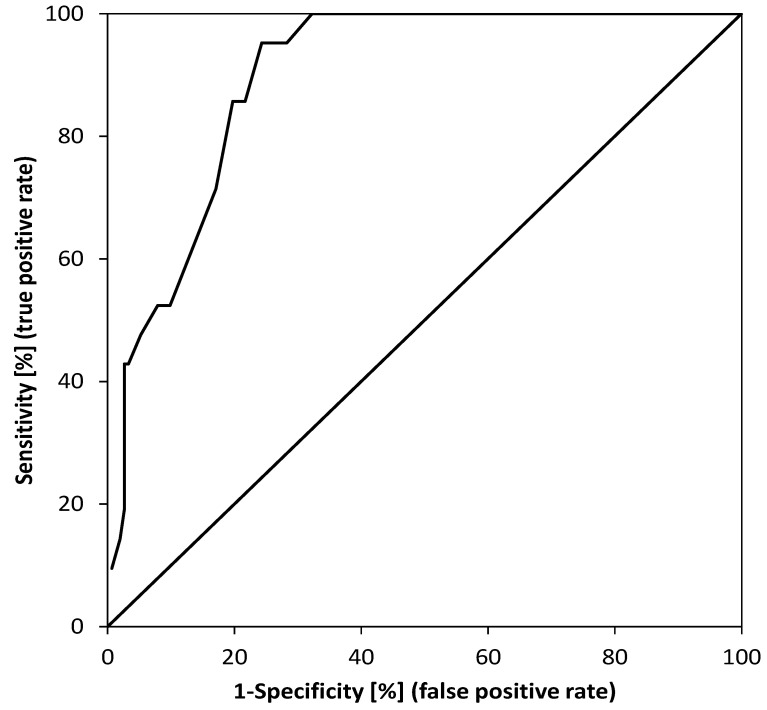
ROC analysis demonstrating the ability to distinguish isoattenuating from hypoattenuating pancreatic carcinomas by tumor size measured on EUS when a cut-off value of ≤ 25.0 mm was used. Sensitivity 95.2% (95% CI 76.2–99.9%), specificity 75.7% (95% CI 68–82.3%), AUC 0.898 (95% CI 0.834–0.939), Youden’s J statistic 0.709; *p* < 0.001.

Based on the size of the pancreatic tumor measured on EUS, the optimal cut off value ≤ 25 mm was determined to discriminate isoattenuating from hypoattenuating pancreatic carcinomas with a sensitivity of 95.2% (95% CI 76.2–99.9%), specificity of 75.7% (95% CI 68–82.3%), AUC of 0.898 (95% CI 0.834–0.939), Youden’s J statistic = 0.709; *p* < 0.001 (Figure 3).

EUS as a reference standard visualized the pancreatic cancer in 100% of patients. In contrast, patients with false negative cytology were included in the study (*n* = 13) of which two were isoattenuating and 11 hypoattenuating on CT. There was no significant difference in EUS-FNA sensitivity (90.5% vs 92.8%; *p* = 0.886) between both tumor groups (Table 4).

**Theorem** **1.** *Dilatation of the pancreatic duct and/or common bile duct and interruption of the pancreatic duct were the most common secondary signs of isoattenuating carcinomas on CT.*

**Theorem** **2.** *EUS-FNA is a highly sensitive method for confirming the diagnosis of isoattenuating pancreatic carcinomas.*

**Theorem** **3.** *According to EUS, all isoattenuating carcinomas were located in pancreatic head with a size up to 30 mm.*

**Theorem** **4.** *Patients with isoattenuating pancreatic carcinomas were resected with curative intent almost twice as often as patients with hypoattenuating carcinomas and, therefore, have higher chance for better prognosis.*

## 4. Discussion

The importance of imaging methods in the diagnosis of pancreatic carcinoma is crucial. In the case of a significant clinical suspicion of pancreatic carcinoma and the absence of a tumor on CT, MRI can be supplemented according to the experience of the specific workplace. EUS biopsy is not considered mandatory before pancreatic surgery in the case of negative CT/MR and high clinical suspicion of pancreatic cancer [5,7,13]. However, if the EUS-FNA is eventually performed and the cytology result is positive, surgery should follow as soon as possible. In the case of negative cytology and persistent significant clinical suspicion of pancreatic carcinoma, surgical exploration of the pancreas is recommended [13,14].

In our study, isoattenuating pancreatic carcinomas on CT occurred in 12.1% of patients with pancreatic cancer, which is comparable to the frequency of 5.4–11% reported in other studies [8,16,17]. In these studies, similar to us, portal and pancreatic phases were used to evaluate the differences in densities on CT; the mean differences in tumor-pancreas densities in these studies were in most cases less than 15 HU, and both patients with and without subsequent surgical resection were included. A slightly higher incidence of isoattenuating carcinomas (14%) evaluated on CT was found in the study of Ishigami et al., who included only patients with pancreatic carcinomas proved by surgical resection [9].

Secondary signs on CT were present in 95.2% (20 of 21) of patients with isoattenuating pancreatic cancer, consistent with 88–100% observed in other studies [8,10]. In one patient without secondary signs on CT, a 10 mm tumor of the pancreatic head was detected by EUS-FNA, which was indicated because of nonspecific abdominal discomfort and newly onset diabetes. The most common signs of isoattenuating pancreatic carcinomas on CT were dilatation of the pancreatic duct or CBD (85.7%) and interruption of the pancreatic duct (76.2%). On the other hand, the occurrence of atrophy of the distal parenchyma and abnormal contour were significantly less common in isoattenuating pancreatic carcinomas (*p* ˂ 0.001 and *p* = 0.01). This can be explained by the fact that isoattenuating pancreatic tumors are usually smaller and less advanced compared to hypoattenuating tumors, and duct changes are also described as an early sign of pancreatic cancer [8,18,19]. Contrary to our results, another described sign of early-stage pancreatic tumor is local atrophy of the pancreas [4,19]. A possible source of this discrepancy can be differences in the definition of pancreatic atrophy, which should be unified in the future. In our study, similar to Aslan, the simplest possible definition of pancreatic atrophy was used [16].

On EUS, all isoattenuating pancreatic carcinomas were located in the head of the pancreas, significantly more often than hypoattenuating carcinomas (100% vs 59.2%, *p* ˂ 0.001). Kim also reported the head of the pancreas to be the most common localization of isoattenuating pancreatic carcinomas (26 out of 30 (87%)), while the incidence in the body and tail of the pancreas was 10% and 3%, respectively [8]. Lower frequency of the isoattenuating tumors in the body or tail of the pancreas can be explained by the fact that tumors in the body and tail tend to be larger (and therefore hypoattenuating) because early clinical symptoms are missing.

In our study, isoattenuating pancreatic carcinomas were significantly smaller on EUS compared to hypoattenuating pancreatic carcinomas (20 vs 31 mm, *p* < 0.001). In the ROC analysis, the optimal cut-off value to discriminate isoattenuating from hypoattenuating pancreatic carcinomas by EUS was ≤ 25 mm (AUC 0.898). In the study by Ishigami et al., the difference in size between iso and hypoattenuating pancreatic carcinomas was even more pronounced (12.4 ± 4.8 mm vs. 30.3 ± 9.0 mm, *p* < 0.0001) [9]. Ishigami, however, determined the size of the tumor on CT in portal venous phase or delayed phase. On the other hand, in another study, the size of all isoattenuating pancreatic carcinomas was in the range of 15–40 mm (median 30 mm) [8]. In contrast to our work, the size of the tumor was measured from surgical specimens and not by imaging methods [8].

In addition, for isoattenuating pancreatic carcinomas, we determined a false negative rate of EUS-FNA, which occurred in two of 21 (9.5%) patients. In both these patients, surgical resection was indicated. The indication for the surgery was the presence of a solid mass seen by EUS in combination with higher levels of oncomarkers and positive cytology from choledochal stenosis obtained by ERCP. EUS-FNA sensitivity was high in both tumor groups with no significant difference (90.5% vs. 92.8%; *p* = 0.886). It is already known that in the case of an indeterminate finding on the pancreas on CT, EUS-FNA is a highly sensitive and accurate method for the detection of a pancreatic neoplasm, especially if the tumor is smaller than 2.0 cm. Wang et al. reported a 92.1% accuracy using EUS in the diagnosis of these tumors [20].

We also found in our study that patients with isoattenuating pancreatic carcinomas were operated on almost twice as often as patients with hypoattenuating pancreatic carcinomas (44.7% vs. 85.7% of patients; *p* = 0.001). Thus, we indirectly confirmed the fact that patients with isoattenuating pancreatic carcinoma have a better prognosis than patients with hypoattenuating pancreatic carcinoma, because surgical treatment of pancreatic carcinomas represents the only potential hope for curing patients with these carcinomas [2,21]. Our study correlates with the study of Kim et al., where almost 86% (30 of 35) of patients with isoattenuating pancreatic carcinoma were surgically resected [8]. Kim also demonstrated longer survival of patients with isoattenuating pancreatic carcinomas compared to patients with hypoattenuating pancreatic carcinomas (median survival 30 months vs. 15.6 months) [8].

A limitation of our study is the retrospective nature of data collection, and inherent bias may be present because it is a single-center study with a small sample size. On the other hand, to our knowledge, this is the third largest study with isoattenuating pancreatic carcinomas, and the only one using EUS-FNA as a reference standard [8,10]. The second possible bias may be the fact that only pancreatic tumors confirmed by EUS-FNA were included. Patients with false positive EUS-FNA were excluded from the study and, therefore, the specificity of EUS-FNA could not be determined. In the study period, only cytological samples were obtained from EUS-FNA, which could not be used for assessing the type and grade of the tumor. Using cytology, it was not possible to determine histological differences in the density of tumor cells, vessels and fibrous tissue in iso and hypoattenuating pancreatic cancer, which may be considered in future studies. The aim of our study was to focus only on patients with a definitive diagnosis of pancreatic cancer, so we could not evaluate the differences in isoattenuating pancreatic carcinomas compared to other diagnoses. To date, only a small number of studies have been performed on the differential diagnosis of isoattenuating pancreatic carcinoma. In a study by Aslan et al., perfusion CT was used to distinguish isoattenuating pancreatic carcinoma from mass-forming pancreatitis [16]. The variability of CT scanners should not affect the measurement of tumor density and the evaluation of secondary signs of the pancreatic tumor.

To conclude, isoattenuating pancreatic head carcinoma can be revealed by indirect signs using CT and confirmed with high sensitivity by EUS-FNA.

## Figures and Tables

**Table 1:** List of inclusion and exclusion criteria of the study.

**Inclusion criteria**
Definite diagnosis of pancreatic carcinoma
Contrast enhanced CT followed by EUS-FNA at the time of the diagnosis
Pancreatic cancer visualized on EUS
Age over 18
**Exclusion criteria**
Incomplete contrast enhanced CT (lack of pancreatic or portal venous phase)
False positive EUS-FNA
Evidence of chronic pancreatitis on CT

**Table 2:** Basic characteristics of isoatenuating and hypoattenuating pancreatic carcinomas.

Variables	Isoattenuating Pancreatic Carcinomas (*n* = 21)	Hypoattenuating Pancreatic Carcinomas (*n* = 152)	*p* Value
Median age (quartiles)	70 (66, 72) years	68 (63.5, 74) years	0.993
Sex (male)	13 (61.9%)	79 (52.0%)	0.534
Surgical resection	18 (85.7%)	68 (44.7%)	0.001

**Table 3:** Frequency of secondary signs of isoatenuating and hypoattenuating pancreatic carcinomas on CT.

Secondary Signs on CT	Isoattenuating Pancreatic Carcinomas (*n* = 21)	Hypoattenuating Pancreatic Carcinomas (*n* = 152)	*p* Value
Abnormal pancreatic contour (n)	7 (33.3%)	99 (65.1%)	0.01
Dilatation of pancreatic duct or CBD (n)	18 (85.7%)	100 (65.8%)	0.11
Atrophy of distal pancreatic parenchyma (n)	2 (9.5%)	77 (50.7%)	˂0.001
Interruption of pancreatic duct (n)	16 (76.2%)	93 (61.2%)	0.27

**Table 4:** Differences between isoatenuating and hypoattenuating pancreatic carcinomas on EUS-FNA.

Variables	Isoattenuating Pancreatic Carcinomas (*n* = 21)	Hypoattenuating Pancreatic Carcinomas (*n* = 152)	*p* Value
**Size on EUS**			
Median (quartiles)	20 (17,23) mm	31 (26,43) mm	˂0.001
Range	10–27 mm	11–94 mm	
**Distribution**			
≤20 mm	11 (52.4%)	12 (7.9%)	
21–30 mm	10 (47.6%)	61 (40.1%)	<0.001
>30 mm	0 (0%)	79 (52.0%)	
**Location on EUS**			
Head	21 (100%)	90 (59.2%)	<0.001
Body	0 (0%)	46 (30.3%)	0.007
Tail	0 (0%)	16 (10.5%)	0.247
**FNA Sensitivity**	19 (90.5%)	141 (92.8%)	0.886

## Data Availability

Not applicable.

## Appendix A. Detailed Description of EUS-FNA Technique

Evaluation of EUS-FNA samples was performed by an experienced cytopathologist using the ROSE method (rapid on-site cytopathology evaluation). After inserting the needle into the target structure and removing the mandren, a negative pressure was applied using a 10 mL syringe. During the application of the negative pressure six passes of the needle in fan-like manner from different areas of the tumor under EUS control of the needle tip were performed. After releasing the negative pressure, the aspiration needle was withdrawn from the echoendoscope and the contents of the needle were placed onto slides. If a sufficient amount of material was taken, it was inserted into a tube with formaldehyde and subsequently examined in a cytoblock.

## Appendix B. Description of CT Scanner Parameters

CT examination was performed on CTs with 16 and 128 rows of detectors (GE LightSpeed 16, GE Healthcare, Milwaukee, WI, USA; Ingenuity 128, Philips Healthcare, Amsterdam, The Netherlands). CT scans using a Phillips Ingenuity 128 were obtained at 120 kVp, dose modulation with maximum 250 mA, pitch of 0.891, and collimation of 128 × 0.625 mm. LightSpeed 16 scanner parameters were 120 kV, 200 mAs, pitch 0.938, rotation time of 0.6 s and collimation of 16 × 1.25 mm. Bolus tracking was used to ascertain the pancreatic phase, which was triggered 15 s after the density in the aorta at the level of the diaphragmatic dome increased by 100 HU. Noncontrast and portal venous phase included the whole abdomen; in pancreatic phase, the scanning span ranged from the diaphragm to the umbilicus level.
